# Radiocatalytic ammonia synthesis from nitrogen and water

**DOI:** 10.1093/nsr/nwae302

**Published:** 2024-08-30

**Authors:** Bo-Shuai Mu, Yang Xu, Zhiyu Tu, Yugang Zhang, Weiqiu Liang, Jiahao Li, Xianglin Wang, Siyong Shen, Junyi Chen, Zhibo Liu

**Affiliations:** Beijing National Laboratory for Molecular Sciences, Radiochemistry and Radiation Chemistry Key Laboratory of Fundamental Science, Key Laboratory of Bioorganic Chemistry and Molecular Engineering of Ministry of Education, College of Chemistry and Molecular Engineering, Peking University, Beijing 100871, China; Beijing National Laboratory for Molecular Sciences, Radiochemistry and Radiation Chemistry Key Laboratory of Fundamental Science, Key Laboratory of Bioorganic Chemistry and Molecular Engineering of Ministry of Education, College of Chemistry and Molecular Engineering, Peking University, Beijing 100871, China; Beijing National Laboratory for Molecular Sciences, Radiochemistry and Radiation Chemistry Key Laboratory of Fundamental Science, Key Laboratory of Bioorganic Chemistry and Molecular Engineering of Ministry of Education, College of Chemistry and Molecular Engineering, Peking University, Beijing 100871, China; Beijing National Laboratory for Molecular Sciences, Radiochemistry and Radiation Chemistry Key Laboratory of Fundamental Science, Key Laboratory of Bioorganic Chemistry and Molecular Engineering of Ministry of Education, College of Chemistry and Molecular Engineering, Peking University, Beijing 100871, China; Beijing National Laboratory for Molecular Sciences, Radiochemistry and Radiation Chemistry Key Laboratory of Fundamental Science, Key Laboratory of Bioorganic Chemistry and Molecular Engineering of Ministry of Education, College of Chemistry and Molecular Engineering, Peking University, Beijing 100871, China; Beijing National Laboratory for Molecular Sciences, Radiochemistry and Radiation Chemistry Key Laboratory of Fundamental Science, Key Laboratory of Bioorganic Chemistry and Molecular Engineering of Ministry of Education, College of Chemistry and Molecular Engineering, Peking University, Beijing 100871, China; Beijing National Laboratory for Molecular Sciences, Radiochemistry and Radiation Chemistry Key Laboratory of Fundamental Science, Key Laboratory of Bioorganic Chemistry and Molecular Engineering of Ministry of Education, College of Chemistry and Molecular Engineering, Peking University, Beijing 100871, China; Beijing National Laboratory for Molecular Sciences, Radiochemistry and Radiation Chemistry Key Laboratory of Fundamental Science, Key Laboratory of Bioorganic Chemistry and Molecular Engineering of Ministry of Education, College of Chemistry and Molecular Engineering, Peking University, Beijing 100871, China; Beijing National Laboratory for Molecular Sciences, Radiochemistry and Radiation Chemistry Key Laboratory of Fundamental Science, Key Laboratory of Bioorganic Chemistry and Molecular Engineering of Ministry of Education, College of Chemistry and Molecular Engineering, Peking University, Beijing 100871, China; Beijing National Laboratory for Molecular Sciences, Radiochemistry and Radiation Chemistry Key Laboratory of Fundamental Science, Key Laboratory of Bioorganic Chemistry and Molecular Engineering of Ministry of Education, College of Chemistry and Molecular Engineering, Peking University, Beijing 100871, China; Peking University-Tsinghua University Center for Life Sciences, Peking University, Beijing 100871, China; Changping Laboratory, Beijing 102206, China; Key Laboratory of Carcinogenesis and Translational Research (Ministry of Education/Beijing), NMPA Key Laboratory for Research and Evaluation of Radiopharmaceuticals (National Medical Products Administration), Department of Nuclear Medicine, Peking University Cancer Hospital & Institute, Beijing 100142, China

**Keywords:** radiocatalysis, γ-ray radiation, N_2_ reduction, NH_3_ synthesis, Ru-based catalysts

## Abstract

The development of alternative methods to the Haber–Bosch process for ammonia (NH_3_) synthesis is a pressing and formidable challenge. Nuclear energy represents a low-carbon, efficient and stable source of power. The harnessing of nuclear energy to drive nitrogen (N_2_) reduction not only allows ‘green’ NH_3_ synthesis, but also offers the potential for the storage of nuclear energy as a readily transportable zero-carbon fuel. Herein, we explore radiocatalytic N_2_ fixation to NH_3_ induced by γ-ray radiation. Hydrated electrons (e^−^_aq_) that are generated from water radiolysis enable N_2_ reduction to produce NH_3_. Ru-based catalysts synthesized by using γ-ray radiation with excellent radiation stability substantially improve NH_3_ production in which the B_5_ sites of Ru particles may play an important role in the activation of N_2_. By benefitting from the remarkable penetrating power of γ-ray radiation, radiocatalytic NH_3_ synthesis can proceed in an autoclave under appropriate pressure conditions, resulting in an NH_3_ concentration of ≤5.1 mM. The energy conversion efficiency of the reaction is as high as 563.7 mg_NH3_·MJ^−1^. This radiocatalytic chemistry broadens the research scope of catalytic N_2_ fixation while offering promising opportunities for converting nuclear energy into chemical energy.

## Teaser

This work provides a radiocatalytic strategy for ammonia synthesis from N_2_ and H_2_O using Ru-based catalysts, creating promising opportunities to convert radiation energy into chemical energy.

## INTRODUCTION

Haber–Bosch industrial ammonia production is considered one of the most significant chemical reactions ever developed [1,2]. Over 50% of global food production relies on ammonia-based fertilizers. However, this process is carried out at a high temperature of ∼500°C and a pressure of ≤20 MPa [3–5]. In addition, it is also energy-intensive and carbon-emitting, generating ∼500 million tons of carbon dioxide (CO_2_) during steam-methane-reforming for hydrogen production [6–8]. To address these issues, electrochemical and photochemical NH_3_ synthesis processes from N_2_ and H_2_O have been developed for sustainable NH_3_ production [9–12]. Inspired by the efforts to develop radiation-induced cleavage chemistry, we are interested in utilizing nuclear energy (e.g. ionizing radiation) to exploit NH_3_ synthesis [13–19].

**Figure 1.**
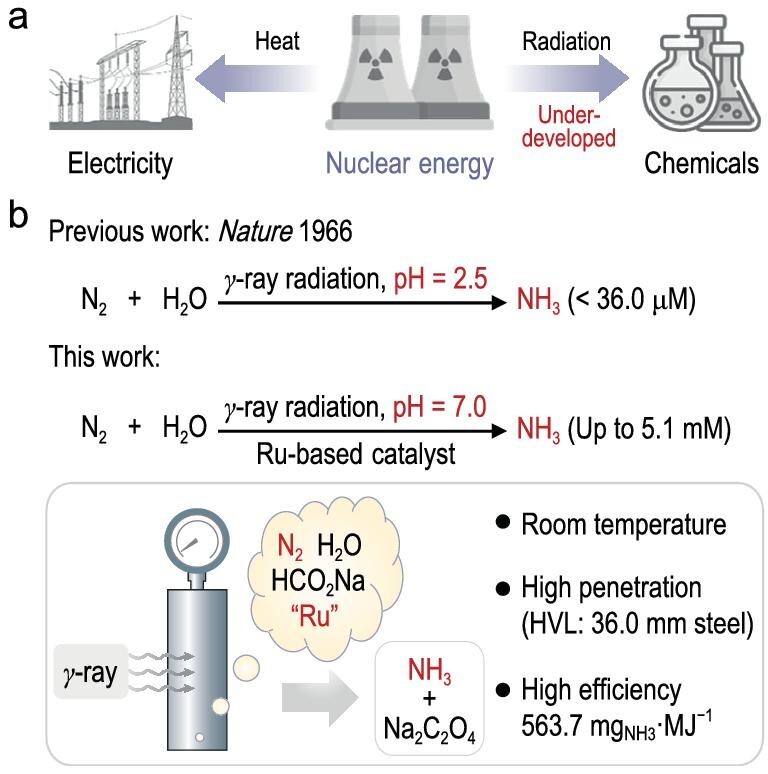
Nuclear energy-induced ammonia synthesis. (a) Conversion of nuclear energy into chemical energy remains to be developed. (b) γ-Ray radiation-induced ammonia synthesis from nitrogen and water. HVL: half-value layer.

Nuclear energy is a carbon-neutral alternative to fossil fuels that may meet the energy demand for baseload electricity and industrial production [20,21]. Ionizing radiation (e.g. γ-rays, X-rays and neutrons) generated from nuclear reactors can be harnessed to enhance the economics of nuclear power, particularly in driving chemical transformations (Fig. 1a) [22–27]. γ-Ray radiation is an appealing option owing to its strong penetrating power (half-value layer: 36.0 mm steel), which enables its use within a certain distance from the radiation source without introducing radioactive contaminants. The radiolysis of water can produce various reductive species, including hydrogen radicals (·H) and hydrated electrons (e^−^_aq_) [28]. In 1966, Getoff *et al.* utilized γ-rays to synthesize NH_3_ from N_2_ and H_2_O at pH 2.5 and achieved an NH_3_ concentration of 36.0 μM [29]. In this case, ·H are the major reductive species produced from water radiolysis. However, the challenge of replicating this work, combined with its low efficiency, has reduced the general interest in exploring this important direction (Fig. 1b).

Herein, we wondered whether hydrated electrons, instead of hydrogen radicals, could drive radiocatalytic N_2_ fixation to produce NH_3_. Hydrated electrons, produced from water radiolysis with a radiolytic yield (expressed as the *G*-value, which is the number of molecules formed by absorbing 100 eV of energy in the system) of 280 nmol·J^−1^ and a standard potential of −2.9 V_NHE_ [30], hold the potential for direct reduction of N_2_ to NH_3_. We synthesized a series of catalysts through the γ-ray radiation-induced reduction method and found that the Ru/SiO_2_ catalyst with excellent radiation stability could substantially improve the NH_3_ production. By benefitting from the remarkable penetration of γ-ray radiation, radiocatalytic NH_3_ synthesis worked well in a thick-wall steel autoclave and the concentration of NH_3_ could reach 5.1 mM. In addition, this radiocatalytic method could achieve a high energy conversion efficiency of 563.7 mg_NH3_·MJ^–1^, surpassing the thermochemical (Haber–Bosch process), photochemical, electrochemical and mechanochemical methods [31–34]. This radiocatalytic chemistry broadens the research scope of catalytic N_2_ fixation while offering promising opportunities for converting nuclear energy into chemical energy (Fig. 1b).

## RESULTS

### Screening of reaction conditions

We started by screening the reaction conditions without the presence of catalysts (Table 1). The major reductive species produced from water radiolysis are pH-dependent (·H when pH < 4.0 and e^−^_aq_ when pH = 4.0–10.0) [35]. Therefore, we first explored the NH_3_ production at pH 2.0 and pH 7.0, when the major reductive species were ·H and e^−^_aq_, respectively. Hydroxyl radicals (·OH) are the major oxidative species that are generated from water radiolysis, which might reduce the efficiency of N_2_ reduction. To quench ·OH, methanol (MeOH), isopropanol (*^i^*PrOH), *tert*-butanol (*^t^*BuOH) and sodium formate (HCO_2_Na) were added as the ·OH scavengers in the nitrogen-saturated water (Table 1, Entries 1–8). It turned out that only the utilization of 10^−3^ M HCO_2_Na at pH 7.0 could enable NH_3_ production with a *G*-value of 0.9 ± 0.2 nmol·J^−1^. This is probably because ·H are more inclined to promote hydrogen (H_2_) evolution reactions rather than nitrogen reduction reactions. When the usage of HCO_2_Na was increased to 1 M, the *G-*value reached 3.0 ± 0.1 nmol·J^−1^ (Table 1, Entry 11). But further increases in the HCO_2_Na usage or the addition of other formates would not give a better result (Table 1, Entries 12–15). We also evaluated the reaction performance at pH 3.0–6.0 and 8.0–12.0, but no further increase in NH_3_ production was observed (Fig. S2). Furthermore, NH_3_ was not detected in the absence of HCO_2_Na, γ-ray radiation or N_2_, suggesting the importance of these components in radiation-induced NH_3_ synthesis (Table 1, Entries 16–18). The main reason why HCO_2_Na can facilitate the reaction is that it can react rapidly with ·OH via hydrogen abstraction (HO· + H − O_2_CNa → [HO···H···O_2_CNa] → H_2_O + ·CO_2_Na, *k* = 3.2 × 10^9^ L·mol^−1^·s^−1^), which can efficiently quench ·OH and prolong the lifetime of e^−^_aq_ [36]. The coumarin trapping experiment further demonstrated that the addition of 1 M HCO_2_Na would cause a decrease of ∼79% in the concentration of ·OH (Fig. S3 and Table S1). In addition, the alkali-metal ions could stabilize negatively charged reactive species e^−^_aq_ through Coulomb interactions to promote the reduction of N_2_ [37].

**Table 1.** Optimization of reaction conditions.

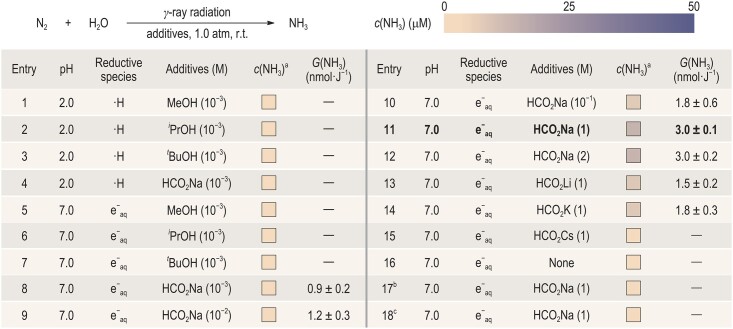

Reaction conditions: γ-ray radiation (250 min, 5000 Gy), *n* = 3; ^a^*c*(NH_3_) was determined via the salicylate method (Fig. S1); ^b^reaction was performed without γ-ray radiation; ^c^reaction was performed using argon (Ar) instead of N_2_.

### Screening of catalysts

Based on the optimized reaction condition, we then focused on evaluating catalysts to improve the efficiency of the radiation-induced NH_3_ synthesis (Table 2). The activation of N_2_ on metal surfaces has been extensively studied in both thermocatalytic and photocatalytic ammonia synthesis [38]. The ability of a metal atom to activate N_2_ depends on the electronic structure of the d-states, where the empty d-orbitals accept the lone-pair electrons from N_2_ (*σ* donation) and occupied d-orbitals back-donate electrons into N_2_ anti-bonding orbitals (*π* back-donation) to weaken the N≡N triple bond [38]. Inspired by previous studies, a series of commercially available typical catalysts have been evaluated in radiation-induced NH_3_ synthesis (characterizations of these catalysts are available in Figs S4–S7). The results showed that only 5% Ru/C and 5% Ru/Al_2_O_3_ could significantly improve the efficiency, with *G-*values of 7.0 ± 1.3 and 10.0 ± 1.4 nmol·J^−1^, respectively (Table 2, Entries 11 and 12). These consequences were consistent with the reported works in which Ru is generally considered to be effective at activating N_2_ [39–41]. However, after one irradiation cycle, the NH_3_ production would be reduced by ∼20%, suggesting undesirable stability of the commercially available 5% Ru/Al_2_O_3_ (Fig. S8). Inductively coupled plasma–optical emission spectroscopy (ICP–OES) measurements have revealed that the Ru loading of 5% Ru/Al_2_O_3_ would decrease to 3.99 wt% after the reaction (Table S2). Therefore, we speculate that the commercially available 5% Ru/Al_2_O_3_ may have radiosensitive sites that lead to the detachment of Ru particles under γ-ray radiation.

**Table 2.** Selected screening of catalysts.

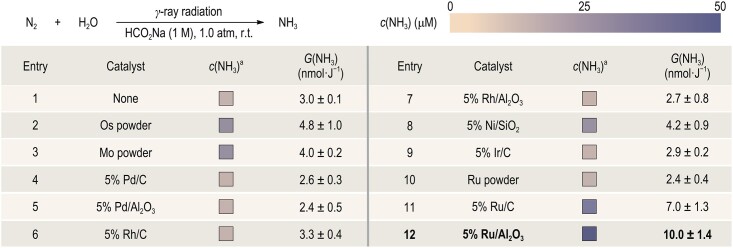

Reaction conditions: γ-ray radiation (250 min, 5000 Gy), *n* = 3; ^a^*c*(NH_3_) was determined via the salicylate method.

### The fabrication and performance of radiation-synthesized catalysts

**Figure 2.**
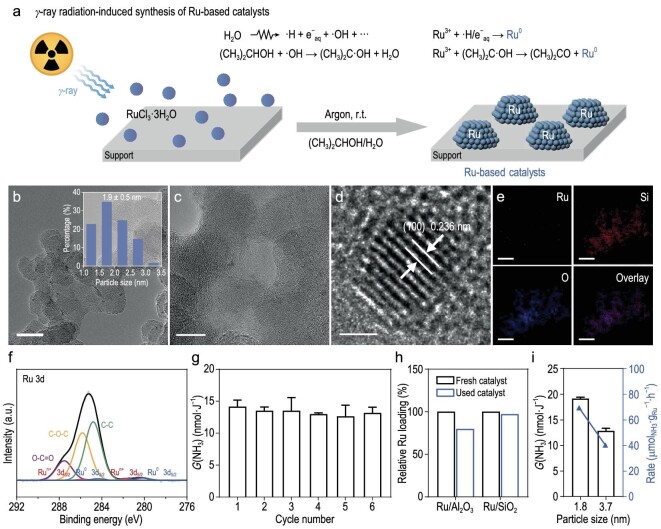
Characterizations and performances of Ru-based catalysts synthesized through the γ-ray radiation-induced reduction method. (a) Schematic illustration of the synthesis process of Ru-based catalysts. (b–d) High-resolution transmission electron microscopy images of Ru/SiO_2_. Inset: Ru particle size distribution of Ru/SiO_2_. Scale bars are 20 nm in (b), 10 nm in (c), 1 nm in (d). (e) Energy dispersive spectroscopy elemental mapping images of Ru/SiO_2_. Scale bars are 100 nm. (f) High-resolution Ru 3d XPS spectrum of Ru/SiO_2_. (g) Cycle test of Ru/SiO_2_ (0.36 wt% Ru loading) in 1 M HCO_2_Na solution with 1.0 atm of nitrogen under 5000 Gy of γ-ray irradiation (*n* = 3). (h) Changes in Ru loadings of commercially available 5% Ru/Al_2_O_3_ and radiation-synthesized Ru/SiO_2_ after receiving 5000 Gy of γ-ray irradiation. (i) *G*(NH_3_) in 1 M HCO_2_Na solution with 1.0 atm of nitrogen under 5000 Gy of γ-ray irradiation using Ru/SiO_2_ with different Ru particle size distributions (*n* = 3). Left: 6.61%-Ru/SiO_2_ with a particle size distribution of 1.8 ± 0.3 nm. Right: 3.7 nm-Ru/SiO_2_ with a particle size distribution of 3.7 ± 0.5 nm.

To address the issue of catalyst deactivation in radiocatalytic reactions, we synthesized Ru-based catalysts through the γ-ray radiation-induced reduction method for better radiation stability. In this method, Ru^3+^ is allowed to be reduced *in situ* to Ru^0^ by reductive species. In addition, the particle size and morphology can be well controlled by tuning the synthesis parameters, such as the dose rate, the absorbed dose and the concentration of the metal precursor [42–45]. Moreover, this process can be carried out under ambient temperature and pressure without extra reductants and initiators. Based on the above advantages, a series of Ru-based catalysts with different supports were obtained by reducing Ru^3+^ in an isopropanol aqueous solution under an argon atmosphere. Isopropanol was used as the ·OH scavenger to ensure the presence of reductive species (e^−^_aq_, ·H and (CH_3_)_2_C·OH) and to facilitate the reduction of Ru^3+^ (Fig. 2a).

High-resolution transmission electron microscopy (HRTEM) images showed that Ru nanoparticles (Ru NPs) of these as-synthesized catalysts were well dispersed on the support, with an average Ru particle size of ∼1.9 nm (Fig. 2b and c, and Fig. S9). In addition, Ru NPs displayed an interplanar spacing of 0.236 nm, which corresponded to the (100) lattice plane (Fig. 2d) [40]. Energy dispersive spectroscopy elemental mapping images further supported the high dispersion of Ru NPs on the surface of the support (Fig. 2e). Powder X-ray diffraction patterns of these as-synthesized catalysts showed that there were no peaks that belonged to Ru NPs, possibly because of low Ru loadings, the small sizes of the Ru NPs and the lack of long-range order in the lattice spacing (Fig. S10). To confirm the valence state of element Ru, X-ray photoelectron spectroscopy (XPS) spectra were measured (Fig. S11). If Ru/SiO_2_ is taken as an example, then the spin-orbital interaction of the 3d orbital made the Ru 3d XPS spectrum exhibit two contributions: 3d_5/2_ and 3d_3/2_. The fitted peaks at 280.2 and 284.4 eV belonged to the Ru^0^ generated from the γ-ray radiation-induced reduction of Ru^3+^, while the fitted peaks at a higher binding energy belonged to the oxidation states of element Ru, which may have come from the partial oxidation of Ru^0^ (Fig. 2f). Similarly, the fitted peaks at 461.9 and 484.2 eV of the Ru 3p XPS spectrum also supported that most of the element Ru was in a metallic state (Fig. S11n) [46,47]. All of the above results demonstrated the successfully controlled fabrication of Ru-based catalysts through the γ-ray radiation-induced reduction method.

Then, these as-synthesized catalysts with different supports were evaluated for ammonia synthesis in the presence of 1 M HCO_2_Na solution with an absorbed dose of 5000 Gy. Among them, Ru/SiO_2_ showed excellent NH_3_ yield, with a usage of 0.50 wt% (Fig. S12). Meanwhile, it is worth noting that the *G*(NH_3_) remained basically unchanged after six irradiation cycles, indicating that Ru/SiO_2_ showed excellent stability (Fig. 2g). HRTEM images, XPS spectra and ICP–OES measurements further revealed that the morphology, valence state, Ru particle size distribution and Ru loading of Ru/SiO_2_ remained nearly unchanged after the reaction (Fig. 2h, Figs S13–S16 and Table S2). We also synthesized Ru/SiO_2_ catalysts with different Ru particle size distributions by tuning the synthesis parameters (characterizations of these catalysts are available in Figs S17–S20). The Ru/SiO_2_ catalyst with a particle size distribution of 1.8 ± 0.3 nm exhibited a significantly better catalytic activity (19.1 ± 0.2 nmol·J^−1^, 69.4 ± 0.7 μmol_NH3_.g_Ru_^−1^.h^−1^) than that with a particle size distribution of 3.7 ± 0.5 nm (12.8 ± 0.4 nmol·J^−1^, 40.1 ± 1.2 μmol_NH3_·g_Ru_^−1^·h^−1^), which could have been related to the active B_5_ sites (Fig. 2i). Previous work showed that the fraction of B_5_ sites is related to the size of the Ru particles, which is most prevalent with an average size of 2.0 nm and decreases significantly with the increasing particle size. In addition, density functional theory calculations showed that step sites enriched with the B_5_ sites are much more reactive for N_2_ dissociation (the activation energy *E*_a_ is 0.4 eV) than the close-packed (001) surface (*E*_a_ is 1.9 eV) [41,48]. Apart from active B_5_ sites, several other step sites and edge sites could also offer competitive reactivity for N_2_ dissociation. Even the quasi-planar ($10{\bar{1}}1$) site exhibits a modest transition state energy, which may contribute to the adsorption and activation of N_2_ [49,50].

### Gamma rays drive radiocatalytic NH_3_ synthesis in a steel autoclave

**Figure 3.**
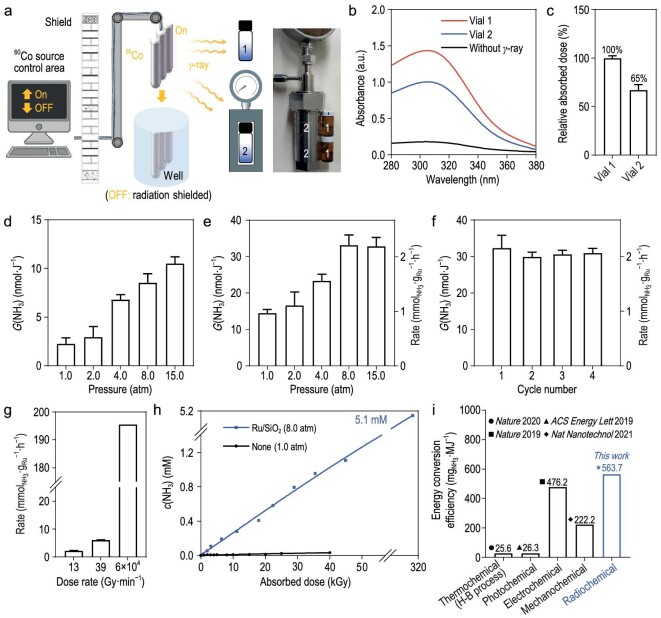
γ-Rays penetrate a steel autoclave and successfully drive radiocatalytic NH_3_ synthesis. (a) Schematic illustration of the radiocatalytic NH_3_ synthesis in the steel autoclave. (b) UV-visible absorption spectra of Fricke dosimeter solutions in Vials 1 and 2 (Vial 1: outside the autoclave, Vial 2: inside the autoclave). (c) The relative absorbed dose of the Fricke dosimeter solutions in Vials 1 and 2 (*n* = 3). *G*(NH_3_) in 1 M HCO_2_Na solution with different nitrogen pressures (d) under 5000 Gy of γ-ray irradiation without catalysts (*n* = 3) and (e) under 3250 Gy of γ-ray irradiation with 0.50 wt% Ru/SiO_2_ (*n* = 3). (f) Cycle test of 0.50 wt% Ru/SiO_2_ in 1 M HCO_2_Na solution with 8.0 atm of nitrogen under 3250 Gy of γ-ray irradiation (*n* = 3). (g) NH_3_ production rates with different absorbed dose rates in 1 M HCO_2_Na solution using Ru/SiO_2_. The pressure is 8.0 atm when the dose rate is 13 or 39 Gy·min^−1^. The pressure is 1.0 atm when the dose rate is 60.0 kGy·min^–1^. (h) Relationship between the absorbed dose and the ammonia production concentration in 1 M HCO_2_Na solution with 8.0 atm of nitrogen using 0.50 wt% Ru/SiO_2_ and with 1.0 atm of nitrogen in the absence of catalysts. For 5.1 mM NH_3_ synthesis, 13.6 mg of HCO_2_Na was added after each absorbed dose of 31.2 kGy. (i) Comparison of ammonia synthesis energy conversion efficiency with different chemical methods.

Encouraged by the superior performance of Ru/SiO_2_ at a pressure of 1.0 atm, we then investigated its performance under different pressures to enhance the mass-transfer efficiency and the solubility of nitrogen. The employment of γ-ray radiation in pressurized reactions is particularly advantageous, as it can directly penetrate a steel autoclave and initiate the reaction. Considering that the wall of the autoclave will cause energy loss, we determined the practical absorbed dose of the solution in the autoclave by using a typical Fricke dosimeter (the mixture solution of (NH_4_)_2_Fe(SO_4_)_2_, NaCl and H_2_SO_4_). During the irradiation, Fe^2+^ can be oxidated to Fe^3+^ by ·OH, H_2_O_2_ and HOO· produced from water radiolysis. The corresponding absorbed dose can be calculated according to the formula by measuring the absorbance of Fe^3+^ at 303 nm [51,52]. Vials 1 and 2 containing Fricke dosimeter solution were irradiated outside and inside the autoclave at the same position within the radiation field for the same irradiation time, resulting in a relative absorbed dose of 65% in Vial 2 compared with Vial 1 (Fig. 3a–c).

The effect of pressure was first evaluated in the presence of 1 M HCO_2_Na without any catalysts. As shown in Fig. 3d, the *G-*value increased gradually from 2.3 ± 0.5 to 10.5 ± 0.6 nmol·J^−1^ when the pressure was increased from 1.0 to 15.0 atm. In the presence of only 0.50 wt% Ru/SiO_2_, elevated pressures could still promote NH_3_ production with a *G-*value of 33.1 ± 2.3 nmol·J^−1^ at a pressure of 8.0 atm. However, the higher pressure had essentially no impact on the *G-*value (Fig. 3e). In addition, an increase or decrease in the usage of Ru/SiO_2_ could not give a better result (Fig. S21). Of note, the catalytic activity remained largely unchanged at a pressure of 8.0 atm after four irradiation cycles, which suggested superior stability and reactivity of the radiation-synthesized Ru/SiO_2_ (Fig. 3f). The rate of NH_3_ synthesis can further significantly increase with the rising dose rate and particularly reach 195.5 mmol_NH3_.g_Ru_^−1^.h^−1^ at an ultra-high dose rate of 60.0 kGy·min^–1^ (irradiated by electron beam) (Fig. 3g). Under these optimized conditions, the relationship between the absorbed dose and the concentration of NH_3_ was studied in the presence of Ru/SiO_2_. It can be seen from Fig. 3h that the relationship between them is positively correlated and the concentration of NH_3_ was 5.1 mM with a total absorbed dose of 312.0 kGy. Compared with the reported thermochemical (Haber–Bosch process) [31], photochemical [32], electrochemical [33] and mechanochemical N_2_ reduction [34], radiocatalytic NH_3_ synthesis exhibited the highest energy conversion efficiency of 563.7 mg_NH3_·MJ^−1^ (Fig. 3i).

### Control experiments and proposed mechanism

**Figure 4.**
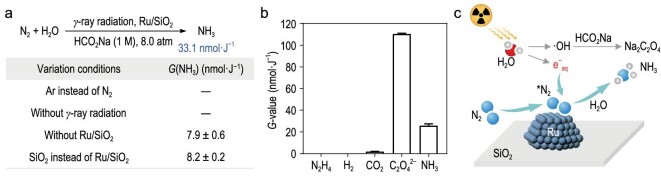
Control experiments and mechanism proposal. (a) Control experiments. These experiments were carried out in 1 M HCO_2_Na solution with 8.0 atm of nitrogen under 3250 Gy of γ-ray irradiation (*n* = 3). (b) Products selectivity. The reactions were carried out under 72.0 kGy of γ-ray irradiation (*n* = 3). The gaseous products (CO_2_ and H_2_) were detected via GC. N_2_H_4_ was detected via UV-visible absorption spectroscopy using the Watt and Chrisp method. The concentration of C_2_O_4_^2−^ was determined via ion chromatography. (c) Proposed mechanism. N_2_ is initially activated on the surface of Ru/SiO_2_ and subsequently reduced to NH_3_ by e^−^_aq_, while C_2_O_4_^2−^ is produced via a free radical coupling of CO_2_^·−^ produced by the hydrogen atom abstraction of HCO_2_^−^.

To confirm that NH_3_ came from N_2_ fixation instead of contaminants or labile *N*-containing compounds, we conducted control experiments to investigate the effect of each reaction component on radiocatalytic NH_3_ synthesis (Fig. 4a). There was no formation of NH_3_ when the nitrogen atmosphere was replaced by an argon atmosphere, eliminating the interference from impurity within the reaction system. Besides, the group without receiving γ-ray irradiation produced negligible NH_3_, indicating that γ-ray radiation was the primary driving force. Furthermore, the *G-*value was found to be only 7.9 ± 0.6 nmol·J^−1^ in the absence of Ru/SiO_2_ or 8.2 ± 0.2 nmol·J^−1^ by using SiO_2_ instead of Ru/SiO_2_, suggesting the critical role of Ru NPs in facilitating the radiocatalytic NH_3_ synthesis. To gain further insights into the reaction mechanism, we detected other species (e.g. N_2_H_4_, H_2_, CO_2_ and C_2_O_4_^2−^) that may have formed during the reaction (Fig. 4b). We first analysed gaseous products by using gas chromatography (GC). A low level of CO_2_ was determined with a *G*-value of 1.8 ± 0.2 nmol·J^−1^ while H_2_ was not detected, which is different from many N_2_ reduction processes in which H_2_ is typically produced as a byproduct. The liquid products were then analysed. N_2_H_4_ was not detected when using the Watt and Chrisp method (Fig. S22). Interestingly, the *G*(C_2_O_4_^2−^) was determined to be 110.5 ± 0.6 nmol·J^−1^ via ion chromatography (Fig. S23), indicating that HCO_2_Na not only acted as a ·OH scavenger to ensure the reactivity of e^−^_aq_, but also offered a potential method for Na_2_C_2_O_4_ synthesis. Based on these results, we proposed a mechanism that N_2_ is initially activated by the surface of Ru/SiO_2_ and subsequently reduced to NH_3_ by e^−^_aq_, while C_2_O_4_^2−^ is produced via a free radical coupling of CO_2_^·−^ generated by the hydrogen atom abstraction of HCO_2_^−^ (Fig. 4c).

## DISCUSSION

The utilization of nuclear energy to power chemical processes is of broad scientific interest. Researchers at Brookhaven National Laboratory attempted to produce fixed nitrogen by using the fission fragment energy in the 1960s. However, the need for specialized fuel and concerns regarding radioactive contamination eventually led to the suspension of the research [22]. In our study, we employed accessible and high-penetrating γ-ray radiation as a form of nuclear energy to avoid the introduction of radioactive contaminants.

In summary, we present radiocatalytic NH_3_ synthesis from N_2_ and H_2_O. Ru-based catalysts synthesized via γ-ray radiation can substantially improve the efficiency of NH_3_ synthesis with a *G*-value of 33.1 ± 2.3 nmol·J^−1^ at a pressure of 8.0 atm. By benefitting from the excellent radiation stability of the Ru/SiO_2_ catalyst, an NH_3_ concentration of 5.1 mM was acquired with an ultra-high absorbed dose of 312.0 kGy. Surprisingly, a high energy conversion efficiency of 563.7 mg_NH3_·MJ^−1^ was achieved by using this radiocatalytic method. In addition, the utilization of HCO_2_Na as the ·OH radical scavenger not only facilitated the radiocatalytic NH_3_ synthesis, but also provided a potential approach for Na_2_C_2_O_4_ synthesis. This radiocatalytic chemistry broadens the research scope of catalytic N_2_ fixation, while offering promising opportunities for converting radiation energy from nuclear waste into chemical energy.

## METHODS

### Preparation of Ru-based catalysts

Ru-based catalysts were prepared through the γ-ray radiation-induced reduction method. By taking Ru/SiO_2_ as an example, 30.0 mg of SiO_2_, 0.15 g of polyvinylpyrrolidone (MW 24 000) and 300 μL of 0.1 M RuCl_3_·3H_2_O aqueous solution were added to a 60-mL glass vial followed by 30 mL of a mixed solution of isopropanol and ultrapure water (3:7, v:v). After stirring and ultrasonication respectively for 20 min, the solution was bubbled with high-purity argon for 20 min to remove oxygen and then sealed for irradiation by ^60^Co γ-rays at a dose rate of 55 Gy·min^−1^ with a total absorbed dose of 49.5 kGy. The catalysts were collected from the solution by using centrifugation at 8000 r/min for 3 min, washed with ultrapure water and ethanol three times respectively and finally obtained after using the freeze-drying process. The same procedure was used for other Ru-based catalysts, such as Ru/TiO_2_, Ru/γ-Al_2_O_3_, Ru/CeO_2_ and Ru/ZnO.

### Radiocatalytic ammonia synthesis

In the general procedure for the radiocatalytic NH_3_ synthesis under 1.0 atm of nitrogen, to a 50-mL Schlenk tube (with a high vacuum value) was added 1 M HCO_2_Na aqueous solution (5 mL) and catalysts (25.0 mg). The tube was evacuated and filled with high-purity nitrogen five times. The reaction suspension was then stirred while being irradiated by ^60^Co γ-rays with a total absorbed dose of 5000 Gy. The resulting solution was filtered and the concentration of NH_3_ was determined via UV-visible absorption spectroscopy using the salicylate method. For the screening of ·OH scavengers, different additives were used instead of HCO_2_Na without the addition of catalysts.

In the general procedure for the radiocatalytic NH_3_ synthesis under low pressures, to a 50-mL autoclave (containing a Teflon tube inside) was added 1 M HCO_2_Na aqueous solution (3 mL) and Ru/SiO_2_ (15.0 mg). The autoclave was purged with high-purity nitrogen. The reaction suspension was then stirred at a low N_2_ pressure while being irradiated by ^60^Co γ-rays. The resulting solution was filtered and the concentration of NH_3_ was determined via UV-visible absorption spectroscopy using the salicylate method. γ-Rays have extremely strong penetration ability and carry high energy. Experiments must strictly follow radiation protection guidelines with extreme care!

### Quantification of NH_3_ via the salicylate method

The quantity of ammonia produced was measured via UV-visible absorption spectroscopy using the salicylate method [53,54]. Typically, sodium salicylate (3.2 g, 20.0 mmol) and NaOH (0.64 g, 16.0 mmol) were dissolved in 50 mL of ultrapure water to form Solution A. Sodium hypochlorite solution (2.5 g) and NaOH (1.5 g, 37.5 mmol) were dissolved in 50 mL of ultrapure water to form Solution B. Then, 0.50 g of sodium nitroprusside dihydrate (Na_2_[Fe(CN)_5_NO]·2H_2_O) was dissolved in 50 mL of ultrapure water to form Solution C. After the reaction, 4 mL of the reaction solution was mixed with 50 μL of Solution A, 500 μL of Solution B and 50 μL of Solution C. The mixture solution was incubated in the dark at 25°C for 60 min. Then, the absorbance at 697 nm of the mixture solution was measured. A series of reference solutions with suitable NH_4_Cl concentrations were created to plot a standard calibration curve. The concentration of ammonia produced after the reaction could be calculated via the calibration curve.

## Supplementary Material

nwae302_Supplemental_File
