# Supplementary material for: Radiocatalytic ammonia synthesis from nitrogen and water
Source: Natl Sci Rev. 2024 Aug 30;11(11):nwae302. doi: 10.1093/nsr/nwae302 (PMC11493089; doi:10.1093/nsr/nwae302)
Supplement: nwae302_Supplemental_File [file nwae302_supplemental_file.pdf]

Supplementary Data for

Radiocatalytic ammonia synthesis from nitrogen and water

Bo-Shuai Mu[1,†], Yang Xu[1,†], Zhiyu Tu[1], Yugang Zhang[1], Weiqiu Liang[1], Jiahao Li[1], Xianglin Wang[1], Siyong Shen[1], Junyi Chen[1] and Zhibo Liu[1,2,3,4,*]

[1]Beijing National Laboratory for Molecular Sciences, Radiochemistry and Radiation Chemistry Key Laboratory of Fundamental Science, Key Laboratory of Bioorganic Chemistry and Molecular Engineering of Ministry of Education, College of Chemistry and Molecular Engineering, Peking University, Beijing 100871, China

[2]Peking University-Tsinghua University Center for Life Sciences, Peking University, Beijing 100871, China

[3]Changping Laboratory, Beijing 102206, China

[4]Key Laboratory of Carcinogenesis and Translational Research (Ministry of Education/Beijing), NMPA Key Laboratory for Research and Evaluation of Radiopharmaceuticals (National Medical Products Administration), Department of Nuclear Medicine, Peking University Cancer Hospital & Institute, Beijing 100142, China

[*]Corresponding author. E-mail: zbliu@pku.edu.cn

[†]Equally contributed to this work.

Materials

All chemicals were used directly without further purification. SiO_2 and ZnO were purchased from Jiangsu XFNANO Materials Tech Co., Ltd. HCO_2Na, Os powder, Ru powder, TiO_2, 5% Ir/C, 5% Ni/SiO_2 and coumarin were purchased from Shanghai Macklin Biochemical Technology Co., Ltd. Polyvinylpyrrolidone (PVP, MW 24000), γ-Al_2O_3 and 5% Ru/C were purchased from Shanghai Aladdin Biochemical Technology Co., Ltd. Mo powder and CeO_2 were purchased from Beijing MREDA Technology Co., Ltd. Formic acid (HCO_2H), potassium formate (HCO_2K), lithium formate (HCO_2Li), cesium formate (HCO_2Cs), sodium salicylate, sodium nitroprusside dihydrate, 5% Ru/Al_2O_3, 5% Pd/C, 5% Pd/Al_2O_3, ruthenium chloride hydrate ($RuCl_3 \cdot 3H_2O$), 7-hydroxycoumarin, *p*-(dimethylamino)benzaldehyde, sodium nitroprusside dihydrate ($Na_2[Fe(CN)_5NO] \cdot 2H_2O$), ammonium iron(II) sulfate ($(NH_4)_2Fe(SO_4)_2$) and hydrazine sulfate were purchased from Bide Pharmatech Co., Ltd. 5% Rh/C and 5% Rh/Al_2O_3 were purchased from Shanghai Haohong Biomedical Technology Co., Ltd. Potassium biphthalate, sodium oxalate ($Na_2C_2O_4$) and sodium hypochlorite solution were purchased from Xilong Scientific Co., Ltd. NaOH, NaCl, NH_4Cl, HCl, methanol (MeOH), ethanol (EtOH), isopropanol (^{i}PrOH), *tert*-butanol (^{t}BuOH), hydrochloric acid and sulfuric acid were purchased from Beijing Tongguang Fine Chemical Company. Ultrapure water was deionized with a Merck Milli-Q® Direct Water Purification System Millipore to a specific resistivity of 18.2 $M\Omega \cdot cm^{-1}$. Nitrogen gas (N_2, 99.999%) and argon gas (Ar, 99.999%) were purchased from Beijing Millennium Capital Gas Co., Ltd.

Characterization

High-resolution transmission electron microscopy (HRTEM) images and energy dispersive spectroscopy (EDS) elemental mapping images were taken on a JEOL JEM-2100F Field Emission Electron Microscope at an acceleration voltage of 200 kV. Powder X-ray diffraction (PXRD) patterns were recorded on a PANalytical X'Pert^3 Powder X-ray Diffractometer with a Cu Kα radiation source (λ = 1.5406 Å). The accelerating voltage and current were 40 kV and 40 mA, respectively. X-ray photoelectron spectroscopy (XPS) spectra were recorded on a Kratos Analytical AXIS Supra X-ray Photoelectron Spectrometer with a monochromatized Al Kα radiation source. XPS spectra were analysed using CasaXPS. The binding energy was calibrated by the C 1s peak at 284.8 eV. UV-visible absorption spectra were measured on a HITACHI U-3010 UV-VIS Spectrophotometer. Fluorescence spectra were measured on a HITACHI F-7000 FL Spectrophotometer. Inductively coupled plasma–optical emission spectroscopy (ICP–OES) measurements were carried out on a Leeman Prodigy 7 ICP–OES. Gas chromatography (GC) measurements were conducted on an Agilent 8890 Gas Chromatography System equipped with a thermal conductivity detector (TCD) and a flame ionization detector (FID). Ion chromatography (IC) measurements were conducted on a Dionex ICS-2000 Ion Chromatography System.

^{60}Co γ-ray irradiation and electron beam irradiation

^{60}Co γ-ray irradiation experiments were carried out in the Department of Applied Chemistry of Peking University. Electron beam irradiation experiments were carried out in Beijing Atom High-Tech Jinhui Radiation Technology Application Co., Ltd.

Determination of G(·OH) in 1 M HCO_2Na solution

The quantity of ·OH in 1 M HCO_2Na solution was measured via fluorescence spectroscopy using an optimized coumarin method [1]. The solution containing 500 μM coumarin and 1 M HCO_2Na was bubbled with Ar for 20 min and then sealed for irradiation by ^{60}Co γ-rays with a total absorbed dose of 46 Gy. After the reaction, 4 mL of the reaction solution was mixed with 4 mL of 50 mM potassium biphthalate solution. The fluorescence spectroscopy of the mixture solution was then measured with the λ_{ex} and λ_{em} at 338 and 454 nm. A series of reference solutions with suitable 7-hydroxycoumarin concentrations were created to plot a standard calibration curve (Fig. S3). The concentration of 7-hydroxycoumarin produced after the reaction could be calculated via the calibration curve. Based on the reaction of ·OH with coumarin, a linear relationship between the G(·OH) and the concentration of 7-hydroxycoumarin after the reaction could be established, and the G(·OH) in 1 M HCO_2Na solution is calibrated based on the G(·OH) in ultrapure water, which is known to be 280.0 nmol·J^{-1} (Table S1).

Preparation of Ru/SiO_2 with a different Ru particle size distribution or Ru loading

To prepare Ru/SiO_2 catalysts with a different Ru particle size distribution or Ru loading, there will be slight adjustments to the synthesis parameters (e.g. the absorbed dose, the concentration of metal precursor $RuCl_3·3H_2O$ and the mass of the capping agent PVP). For 3.7 nm-Ru/SiO_2, the capping agent PVP was not added. For 0.76%-Ru/SiO_2, the total absorbed dose was adjusted to 231.0 kGy. For 2.82%-Ru/SiO_2, the concentration of metal precursor $RuCl_3·3H_2O$ was adjusted to 1 M. For 6.61%-Ru/SiO_2, the concentration of $RuCl_3·3H_2O$ was adjusted to 1 M and the mass of PVP was adjusted to 30.0 mg.

Recyclability tests

After the previous run, the catalysts were collected from the solution by using centrifugation at 8000 rpm for 3 min, washed with ultrapure water three times and finally obtained after using the freeze-drying process. The catalysts were then used for the next cycle.

Quantification of N_2H_4 via the Watt and Chrisp method

The quantity of hydrazine produced was measured via UV-visible absorption spectroscopy using the Watt and Chrisp method [2]. Typically, 4.0 g of *p*-(dimethylamino)benzaldehyde was dissolved in the mixture of 20 mL of concentrated hydrochloric acid and 200 mL of ethanol to form the color reagent. After the reaction, 1 mL of the reaction solution was mixed with 5 mL of color reagent and then diluted to 25 mL with 0.12 M hydrochloric acid aqueous solution. The mixture solution was incubated in the dark at 25 ℃ for 20 min. Then, the absorbance at 458

nm of the mixture solution was measured. A series of reference solutions with suitable hydrazine sulfate concentrations were created to plot a standard calibration curve. The concentration of hydrazine produced after the reaction could be calculated via the calibration curve (Fig. S22).

Quantification of $C_2O_4^{2-}$ via ion chromatography

After the reaction, the reaction solution was diluted and then analysed via ion chromatography with a conductivity detector. A series of reference solutions with suitable $C_2O_4^{2-}$ concentrations were created to plot a standard calibration curve. The concentration of $C_2O_4^{2-}$ produced after the reaction could be calculated via the calibration curve (Fig. S23).

Determination of the absorbed dose in the autoclave

In a typical process, 2 mL of the Fricke dosimeter solution, containing 10^{-3} M ammonium iron(II) sulfate and 10^{-3} M sodium chloride in 0.4 M sulfuric acid, was added to a 5 mL glass vial [3]. Then, the glass vial was placed in the autoclave and irradiated at a specific location within the radiation field for a specific reaction time. After the reaction, the absorbance at 303 nm of the mixture solution was measured. The absorbed dose of the Fricke dosimeter solution in the autoclave can be calculated according to the formula below. To determine the loss of radiation energy after γ-ray passes through the autoclave, another glass vial containing Fricke dosimeter solution was placed outside the autoclave and irradiated at the same location within the radiation field for the same reaction time. The absorbed dose was also calculated according to the formula below. The difference in absorbance at 303 nm between samples with and without irradiation is recorded as ΔA.

Calculation of the absorbed dose

The absorbed dose of the Fricke dosimeter solution is calculated by the following formula:

$$D_F = N_A \times \Delta A \times 100/(\Delta\varepsilon\rho l \times 10^3 G(Fe^{3+}) \times f)\ (Gy)$$

where D_F is the absorbed dose calibrated using the Fricke dosimeter, N_A is Avogadro's constant (6.02×10^{23}), ΔA is the difference in absorbance at 303 nm between samples with and without irradiation, $\Delta\varepsilon$ is the difference in molar absorption coefficient between samples with and without irradiation and has a value of 2204 L (mol·cm)$^{-1}$, ρ has a value of 1.0245 g·mL^{-1}, $G(Fe^{3+})$ has a value of 15.5, f is the unit conversion factor and has a value of 6.242×10^{15}, and l is the optical path length and has a value of 1 cm.

Discussion about the absorbed dose of catalysts

The absorbed dose (D) is related to the mass energy-absorption coefficient (μ_{en}/ρ) of the matter. D is calculated by the following formula [4]:

$$D = 8.76 \times 10^3 \cdot X \cdot (\mu_{en}/\rho)/(\mu_{en}/\rho)_{air}$$

where X is the exposure of a specific position within the radiation field, μ_{en}/ρ is the mass energy-absorption coefficient of the sample, $(\mu_{en}/\rho)_{air}$ is the mass

energy-absorption coefficient of the air. For ^{60}Co γ-rays, the Compton effect is the main interaction between γ-rays and the matter. Therefore, $(\mu_{en}/\rho)/(\mu_{en}/\rho)_{air}$ can be approximated by $(Z/A)/(Z/A)_{air}$, where Z/A is the ratio of the atomic number of the matter to its relative atomic mass. Hence, the formula can be rewritten as follows:

$$D = 8.76 \times 10^3 \cdot X \cdot (Z/A)/(Z/A)_{air}$$

For example, the Z/A of water is $(2 \times 1 + 8)/18.02 \approx 0.55$, the Z/A of Ru is $44/101.07 \approx 0.44$, and the Z/A of SiO_2 is $(2 \times 8 + 14)/60.08 \approx 0.50$. Considering the proportions of the different components in the reaction solution (25.0 mg 0.36%-Ru/SiO_2 in 5 mL 1 M HCO_2Na aqueous solution), the average of Z/A is approximated as $0.55 \times 5/5.025 + 0.44 \times 0.00009/5.025 + 0.50 \times 0.02491/5.025 \approx 0.55$, which is close to the Z/A of water.

Therefore, the absorbed dose of the catalysts can be neglected.

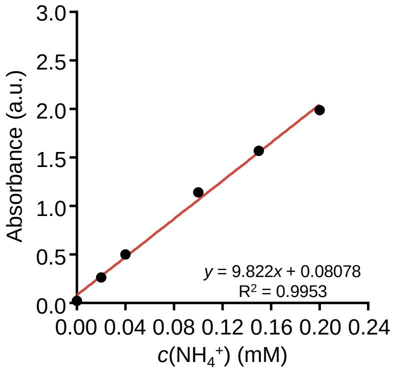

Fig. S1 The calibration curve for quantifying ammonia via the salicylate method.

$$N_2 + H_2O \xrightarrow[\text{additives, 1.0 atm, r.t.}]{\gamma\text{-ray radiation}} NH_3$$

$c(NH_3)$ (μM)

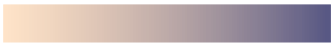

Entry	pH	Additives (M)	$c(NH_3)$	$G(NH_3)$ (nmol·J^{-1})
1	3.0	HCO_2Na (1) + HCO_2H		—
2	4.0	HCO_2Na (1) + HCO_2H		—
3	5.0	HCO_2Na (1) + HCO_2H		0.9 ± 0.2
4	6.0	HCO_2Na (1) + HCO_2H		2.8 ± 0.3
**5**	**7.0**	**HCO_2Na (1)**		**3.0 ± 0.1**
6	8.0	HCO_2Na (1) + NaOH		2.5 ± 0.2
7	9.0	HCO_2Na (1) + NaOH		2.0 ± 0.4
8	10.0	HCO_2Na (1) + NaOH		1.0 ± 0.2
9	11.0	HCO_2Na (1) + NaOH		—
10	12.0	HCO_2Na (1) + NaOH		—

Fig. S2 $G(NH_3)$ in 1 M HCO_2Na solution with 1.0 atm of nitrogen under 5000 Gy of γ-ray irradiation with different pH values (n = 3). HCO_2H and NaOH were used to adjust the pH value.

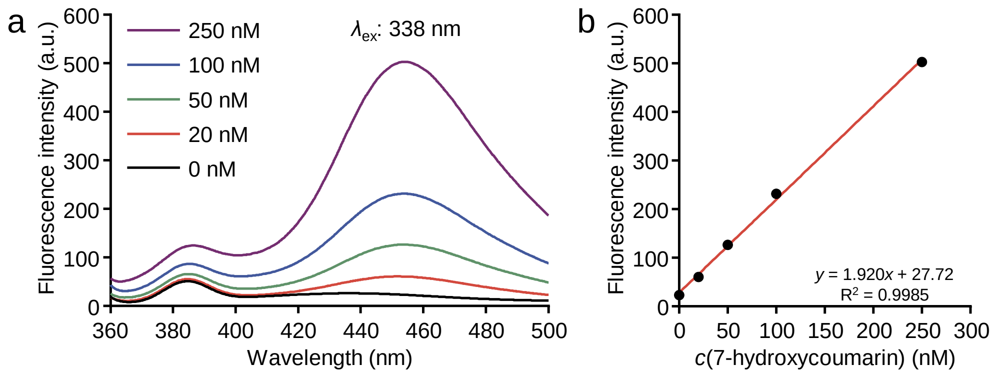

Fig. S3 (a) Fluorescence spectra of different concentrations of 7-hydroxycoumarin. (b) The calibration curve between the fluorescence intensities at 454 nm and the concentrations of 7-hydroxycoumarin.

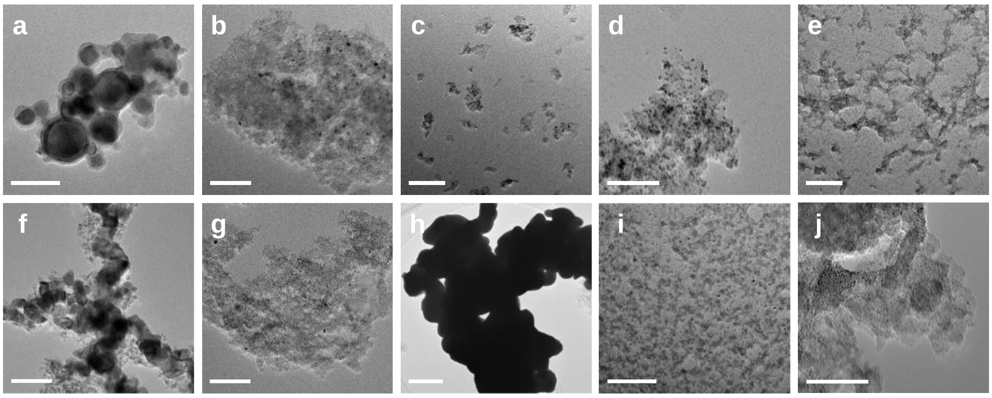

Fig. S4 High-resolution transmission electron microscopy images of commercially available catalysts in Table 2. (a) Mo powder, (b) 5% Pd/C, (c) 5% Pd/Al_2O_3, (d) 5% Rh/C, (e) 5% Rh/Al_2O_3, (f) 5% Ni/SiO_2, (g) 5% Ir/C, (h) Ru powder, (i) 5% Ru/C, (j) 5% Ru/Al_2O_3. Scale bars are 100 nm in (a–c), (e–g), (i), 50 nm in (d), (j), 1 μm in (h).

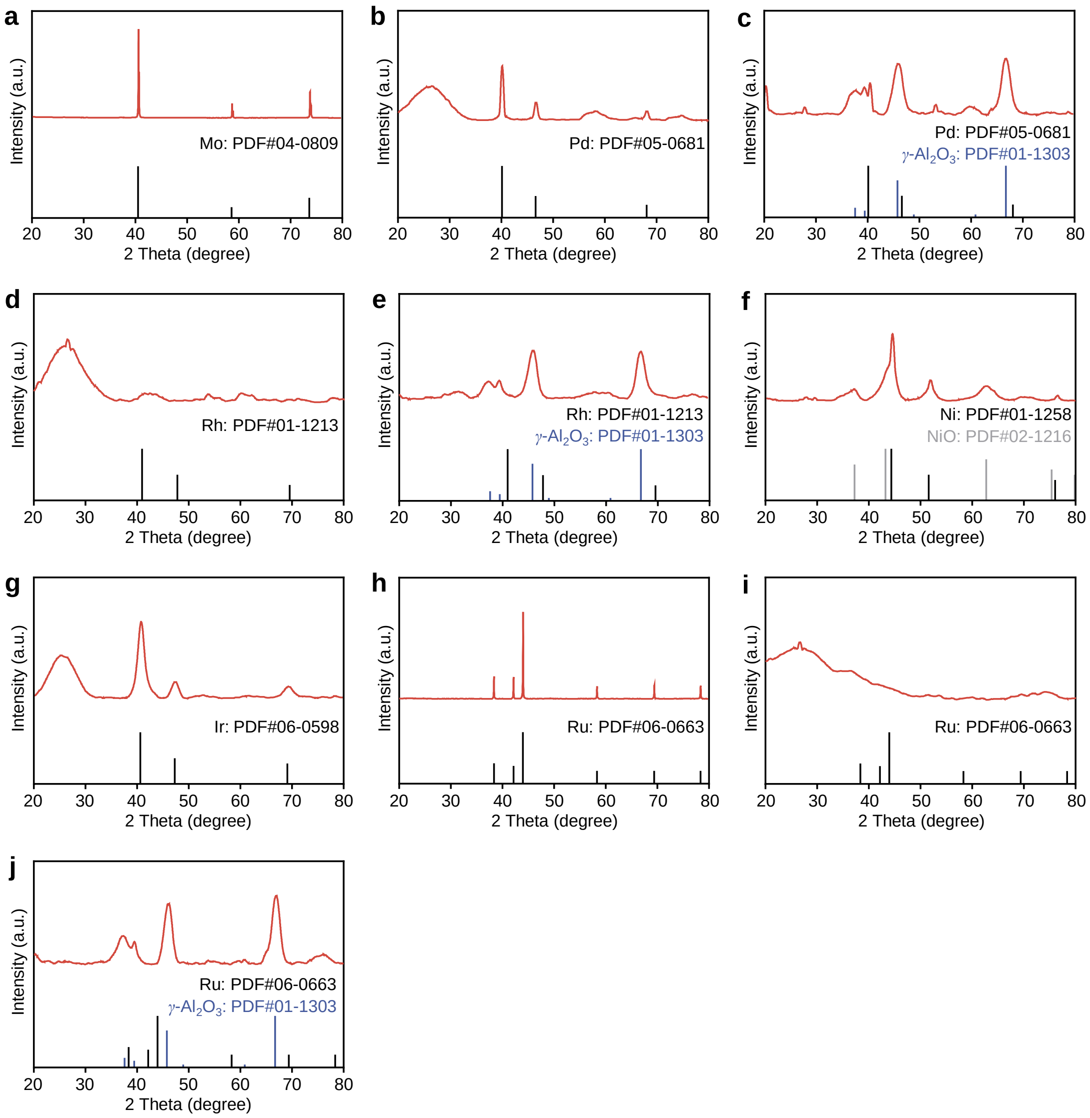

Fig. S5 Powder X-ray diffraction patterns of commercially available catalysts in Table 2. (a) Mo powder, (b) 5% Pd/C, (c) 5% Pd/Al_2O_3, (d) 5% Rh/C, (e) 5% Rh/Al_2O_3, (f) 5% Ni/SiO_2, (g) 5% Ir/C, (h) Ru powder, (i) 5% Ru/C, (j) 5% Ru/Al_2O_3.

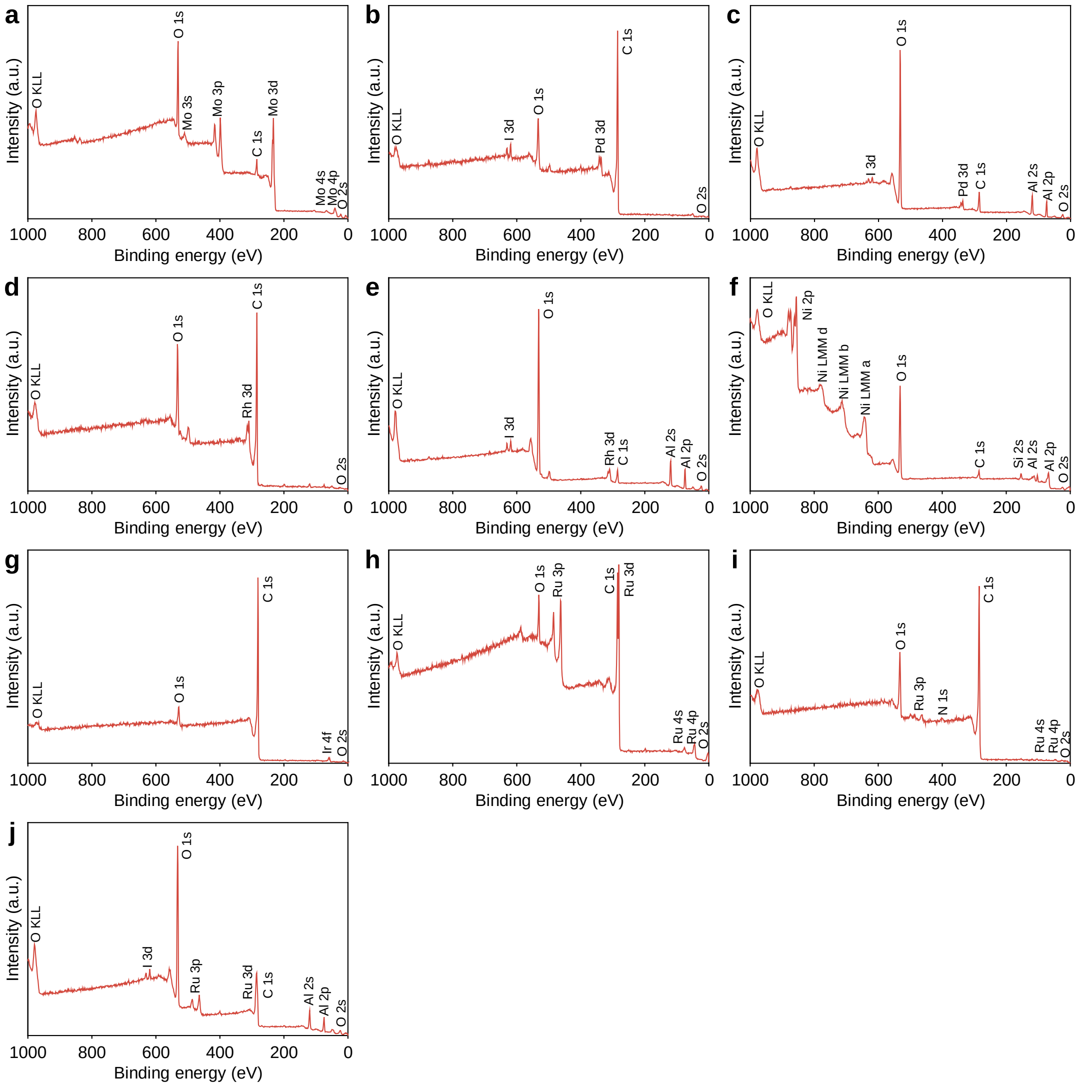

Fig. S6 X-ray photoelectron spectroscopy survey spectra of commercially available catalysts in Table 2. (a) Mo powder, (b) 5% Pd/C, (c) 5% Pd/Al_2O_3, (d) 5% Rh/C, (e) 5% Rh/Al_2O_3, (f) 5% Ni/SiO_2, (g) 5% Ir/C, (h) Ru powder, (i) 5% Ru/C, (j) 5% Ru/Al_2O_3.

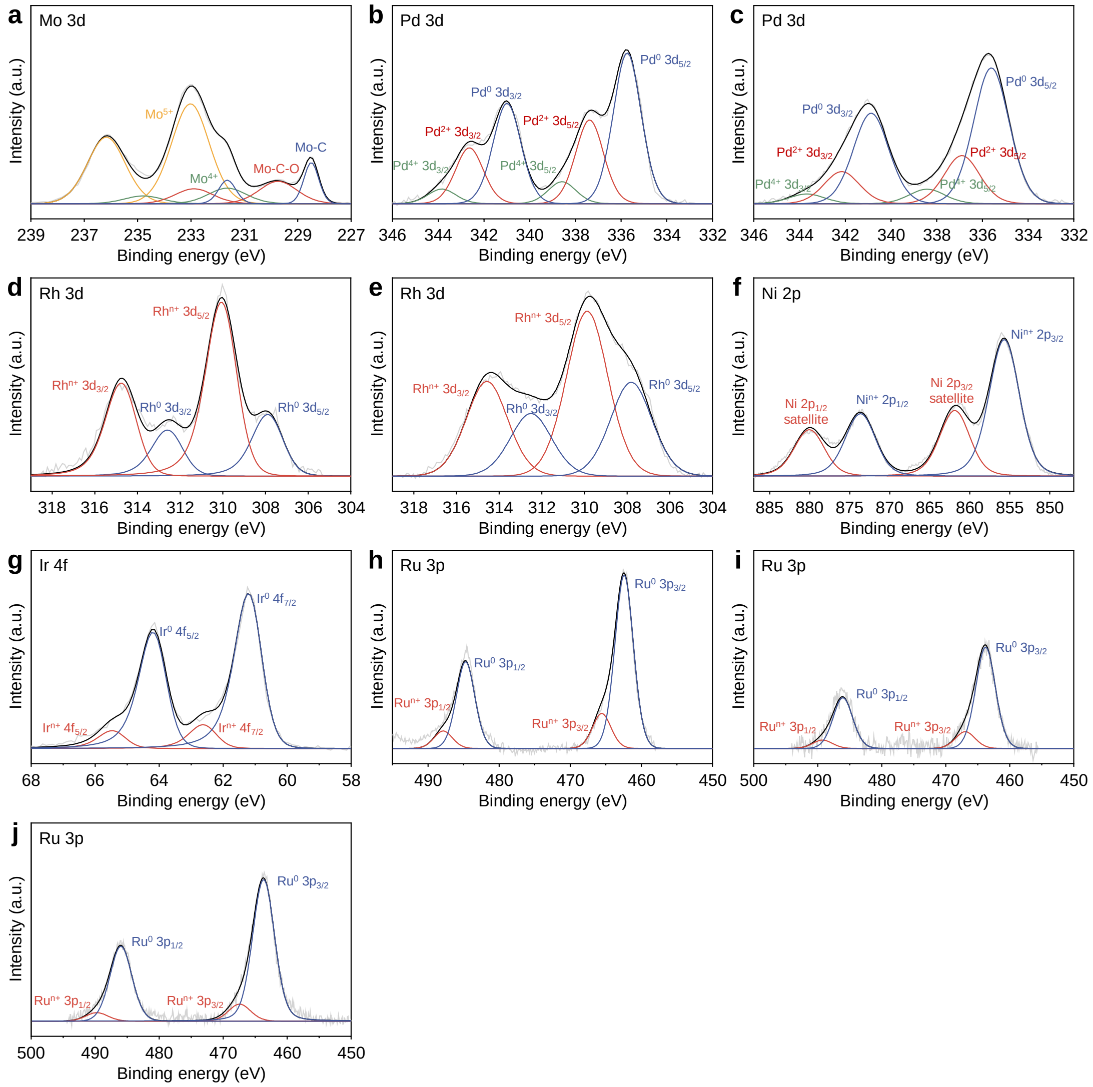

Fig. S7 High-resolution X-ray photoelectron spectroscopy spectra of commercially available catalysts in Table 2. (a) High-resolution Mo 3d XPS spectrum of Mo powder. (b) High-resolution Pd 3d XPS spectrum of 5% Pd/C. (c) High-resolution Pd 3d XPS spectrum of 5% Pd/Al_2O_3. (d) High-resolution Rh 3d XPS spectrum of 5% Rh/C. (e) High-resolution Rh 3d XPS spectrum of 5% Rh/Al_2O_3. (f) High-resolution Ni 2p XPS spectrum of 5% Ni/SiO_2. (g) High-resolution Ir 4f XPS spectrum of 5% Ir/C. (h) High-resolution Ru 3p XPS spectrum of Ru powder. (i) High-resolution Ru 3p XPS spectrum of 5% Ru/C. (j) High-resolution Ru 3p XPS spectrum of 5% Ru/Al_2O_3.

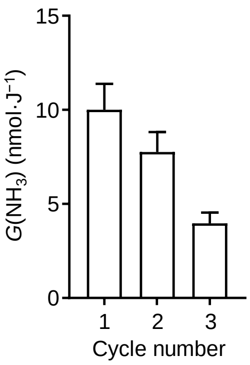

Fig. S8 Cycle test of commercially available 5% Ru/Al_2O_3 in 1 M HCO_2Na solution with 1.0 atm of nitrogen under 5000 Gy of *γ*-ray irradiation (*n* = 3).

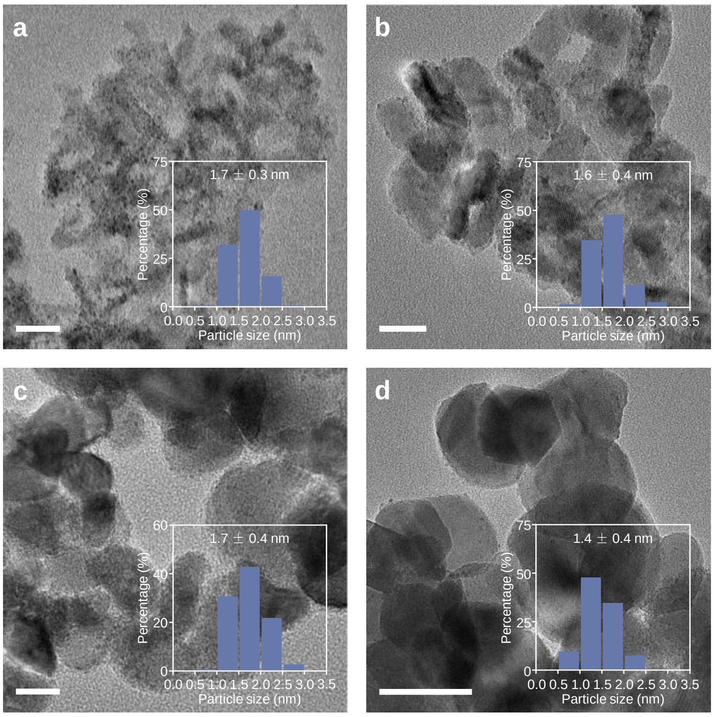

Fig. S9 High-resolution transmission electron microscopy images and corresponding Ru particle size distributions of radiation-synthesized Ru-based catalysts. (a) Ru/γ-Al_2O_3, (b) Ru/TiO_2, (c) Ru/CeO_2, (d) Ru/ZnO. Scale bars are 20 nm in (a–c), 50 nm in (d).

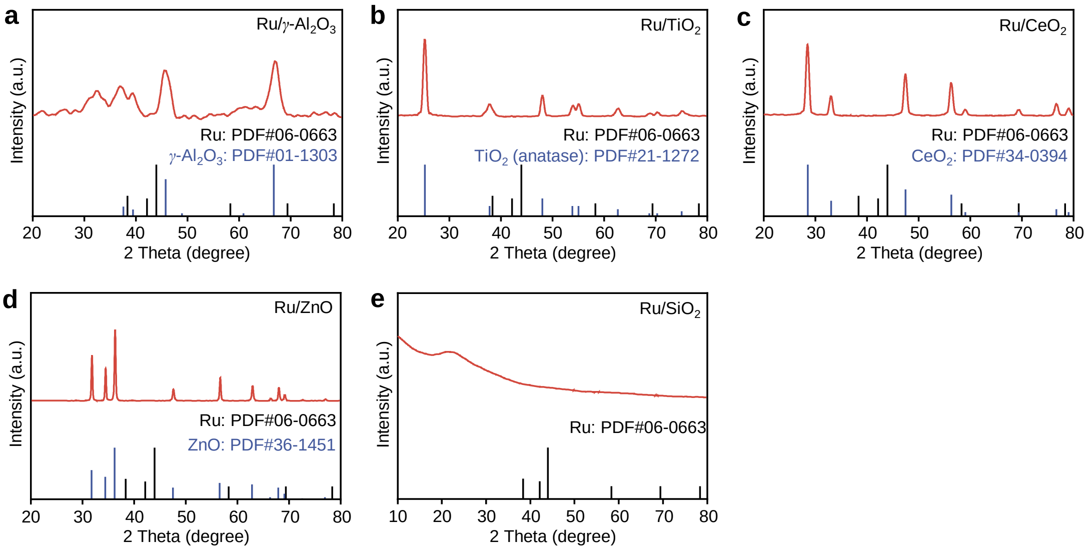

Fig. S10 Powder X-ray diffraction patterns of radiation-synthesized Ru-based catalysts. (a) Ru/γ-Al_2O_3, (b) Ru/TiO_2, (c) Ru/CeO_2, (d) Ru/ZnO, (e) Ru/SiO_2.

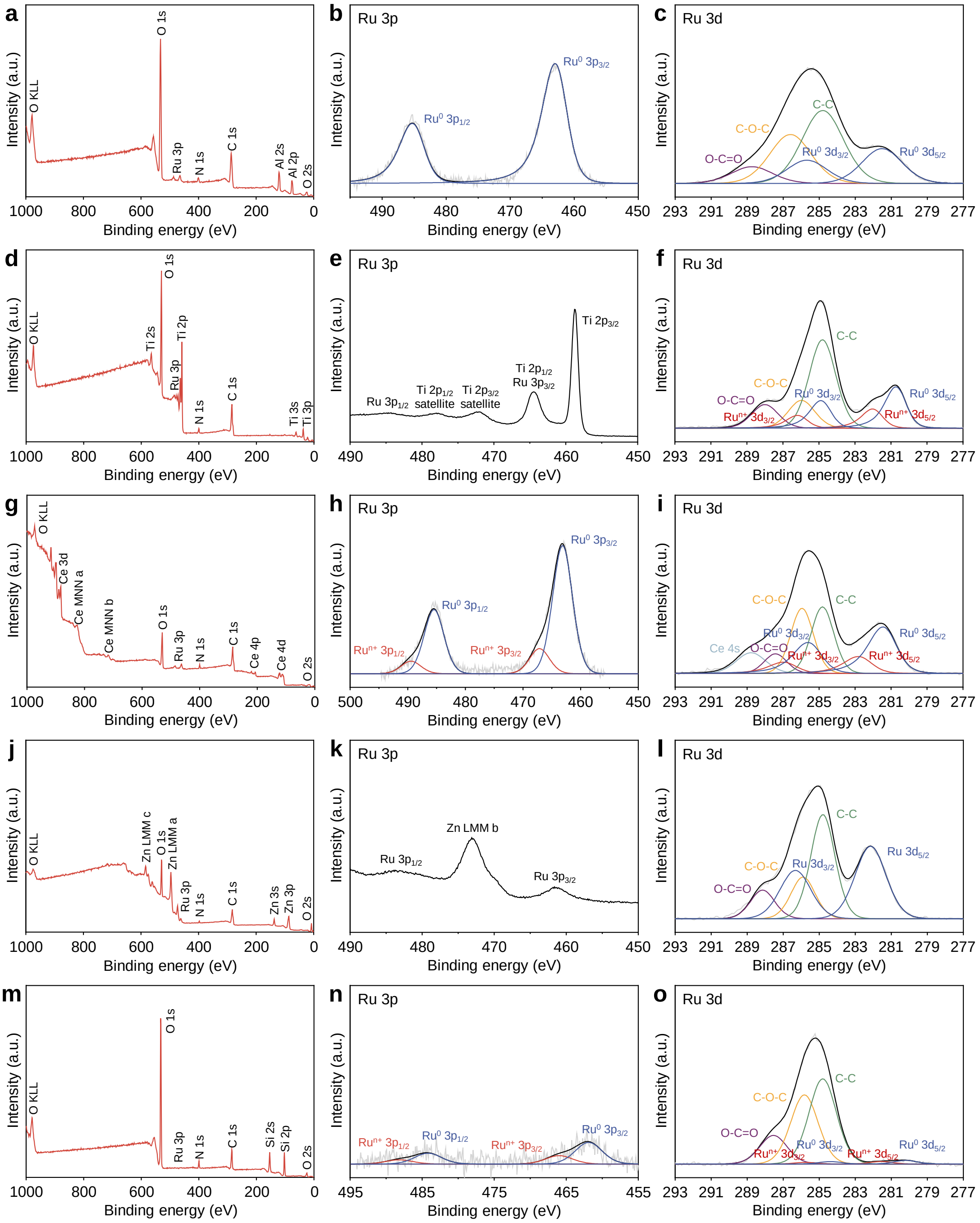

Fig. S11 X-ray photoelectron spectroscopy survey spectra and high-resolution Ru 3p and Ru 3d XPS spectra of radiation-synthesized Ru-based catalysts. (a–c) Ru/γ-Al_2O_3, (d–f) Ru/TiO_2, (g–i) Ru/CeO_2, (j–l) Ru/ZnO, (m–o) Ru/SiO_2.

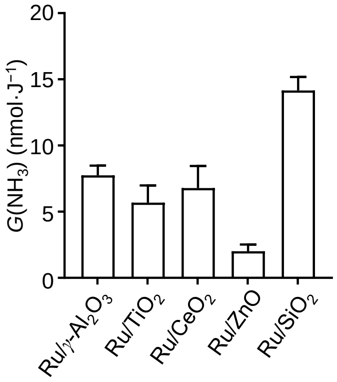

Fig. S12 $G(NH_3)$ in 1 M HCO_2Na solution with 1.0 atm of nitrogen under 5000 Gy of γ-ray irradiation using radiation-synthesized Ru-based catalysts (n = 3).

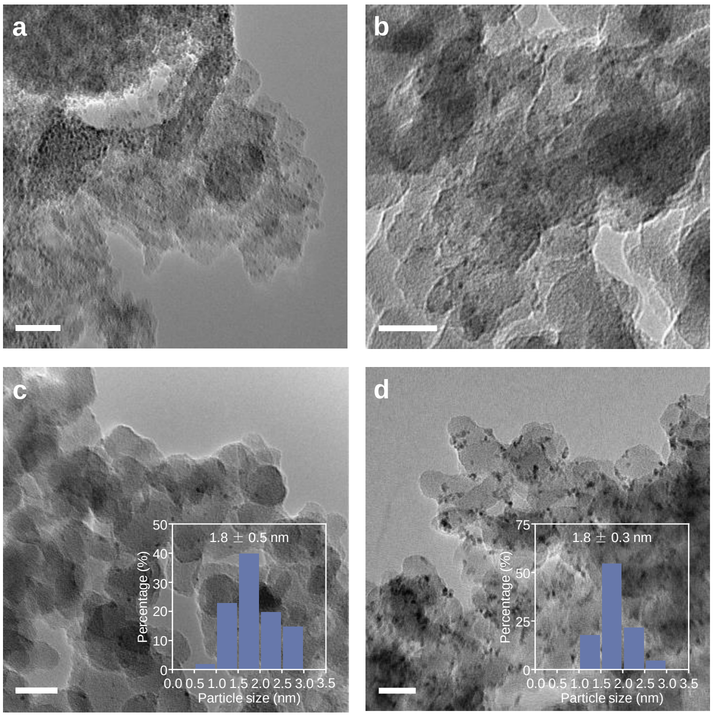

Fig. S13 High-resolution transmission electron microscopy images of (a) fresh commercially available 5% Ru/Al_2O_3, (b) used commercially available 5% Ru/Al_2O_3, (c) used Ru/SiO_2 and (d) used 6.61%-Ru/SiO_2. Scale bars are 20 nm.

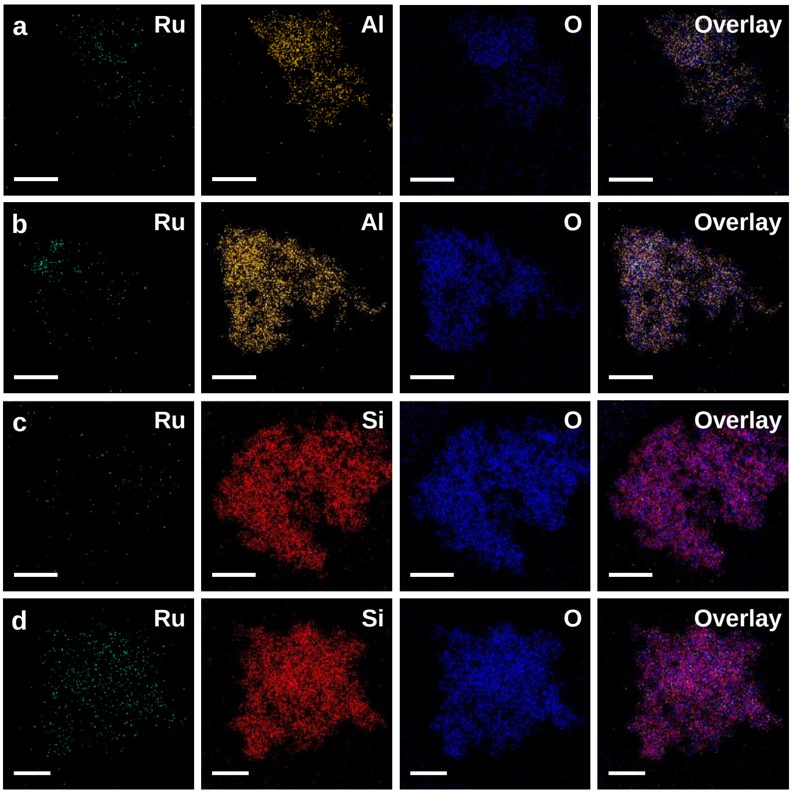

Fig. S14 Energy dispersive spectroscopy elemental mapping images of (a) fresh commercially available 5% Ru/Al_2O_3, (b) used commercially available 5% Ru/Al_2O_3, (c) used Ru/SiO_2 and (d) used 6.61%-Ru/SiO_2. Scale bars are 100 nm in (a), (b), (d), 250 nm in (c).

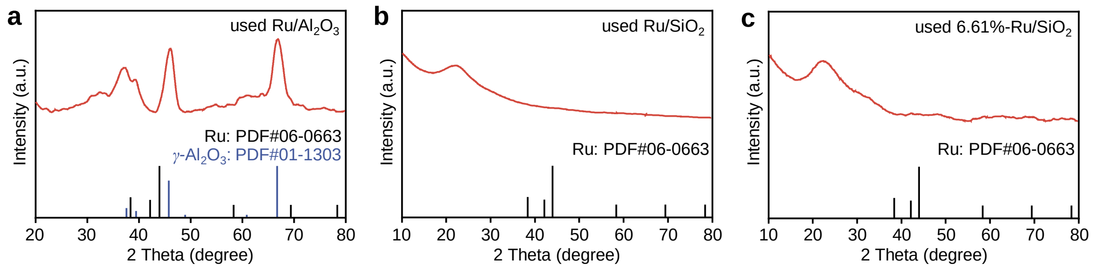

Fig. S15 Powder X-ray diffraction patterns of (a) used commercially available 5% Ru/Al_2O_3, (b) used Ru/SiO_2 and (c) used 6.61%-Ru/SiO_2.

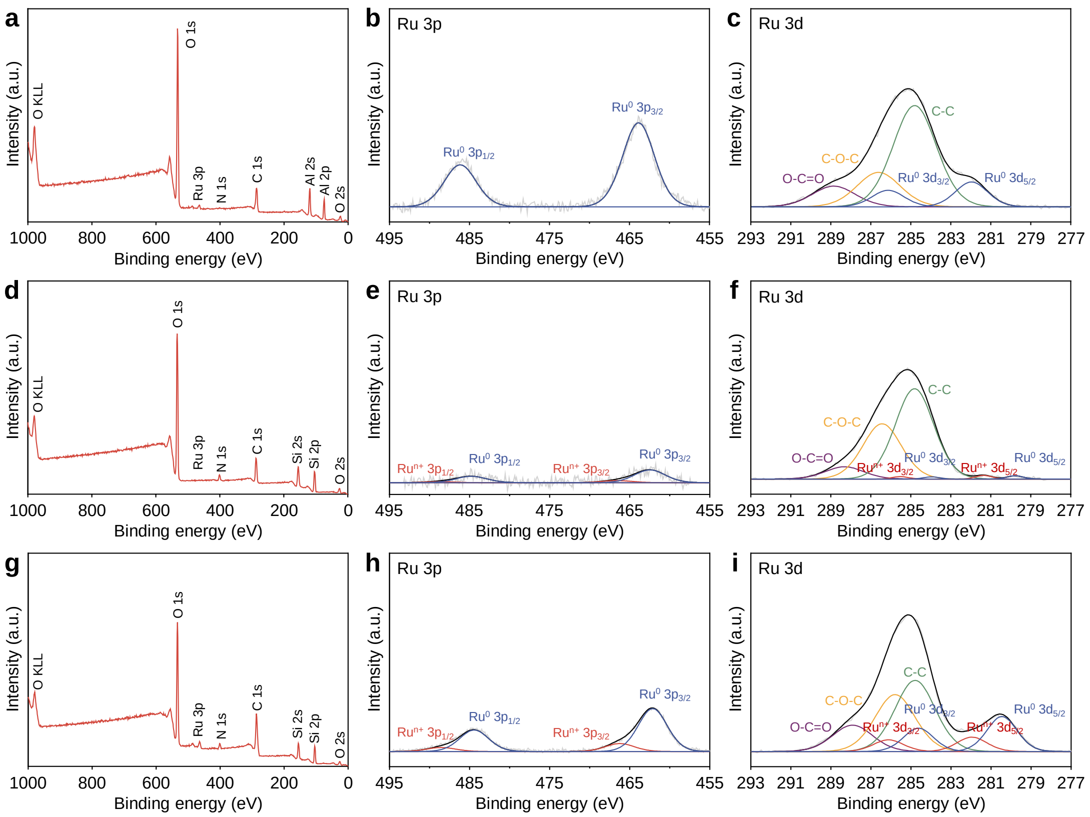

Fig. S16 X-ray photoelectron spectroscopy survey spectra and high-resolution Ru 3p and Ru 3d XPS spectra of (a–c) used commercially available 5% Ru/Al_2O_3, (d–f) used Ru/SiO_2 and (g–i) used 6.61%-Ru/SiO_2.

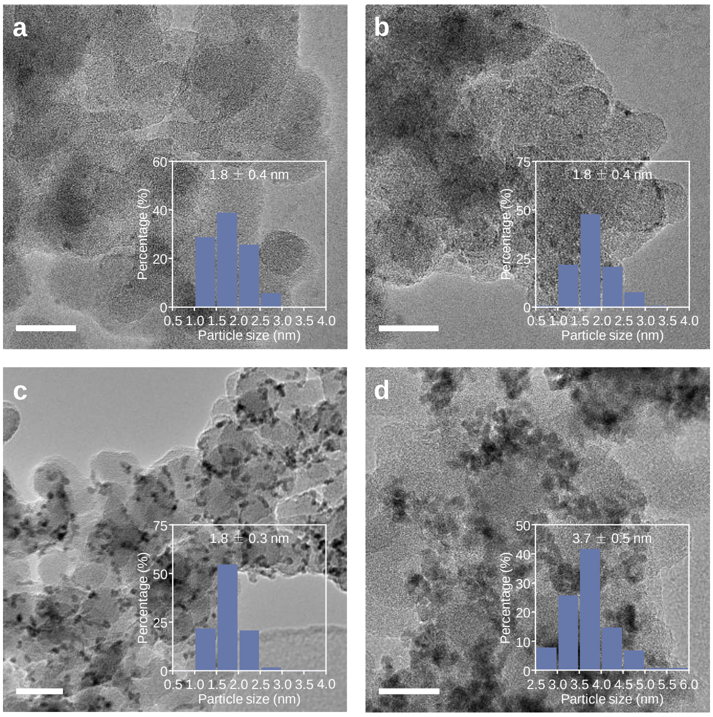

Fig. S17 High-resolution transmission electron microscopy images and corresponding Ru particle size distributions of Ru/SiO_2 with a different Ru loading or Ru particle size distribution. (a) 0.76%-Ru/SiO_2, (b) 2.82%-Ru/SiO_2, (c) 6.61%-Ru/SiO_2, (d) 3.7 nm-Ru/SiO_2. Scale bars are 20 nm.

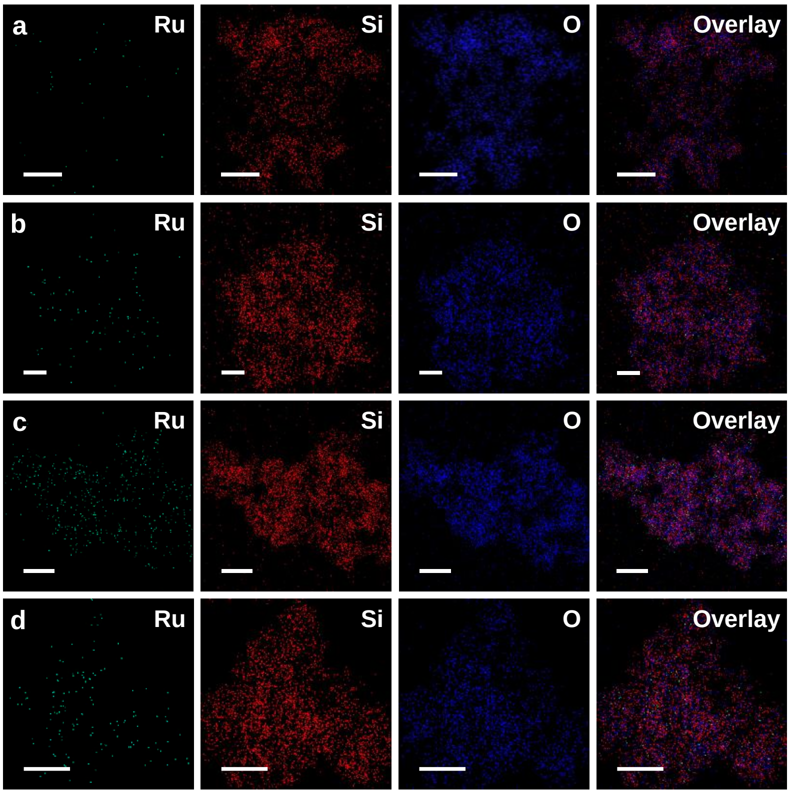

Fig. S18 Energy dispersive spectroscopy elemental mapping images of Ru/SiO_2 with a different Ru loading or Ru particle size distribution. (a) 0.76%-Ru/SiO_2, (b) 2.82%-Ru/SiO_2, (c) 6.61%-Ru/SiO_2, (d) 3.7 nm-Ru/SiO_2. Scale bars are 100 nm in (a–c), 250 nm in (d).

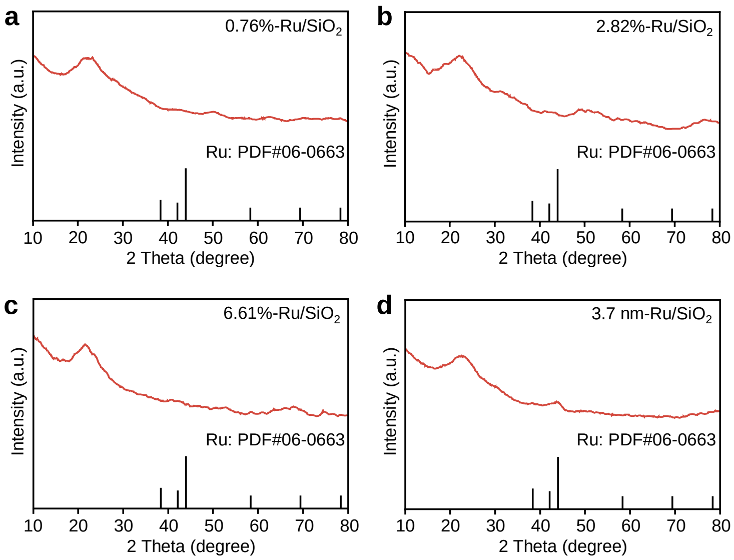

Fig. S19 Powder X-ray diffraction patterns of Ru/SiO_2 with a different Ru loading or Ru particle size distribution. (a) 0.76%-Ru/SiO_2, (b) 2.82%-Ru/SiO_2, (c) 6.61%-Ru/SiO_2, (d) 3.7 nm-Ru/SiO_2.

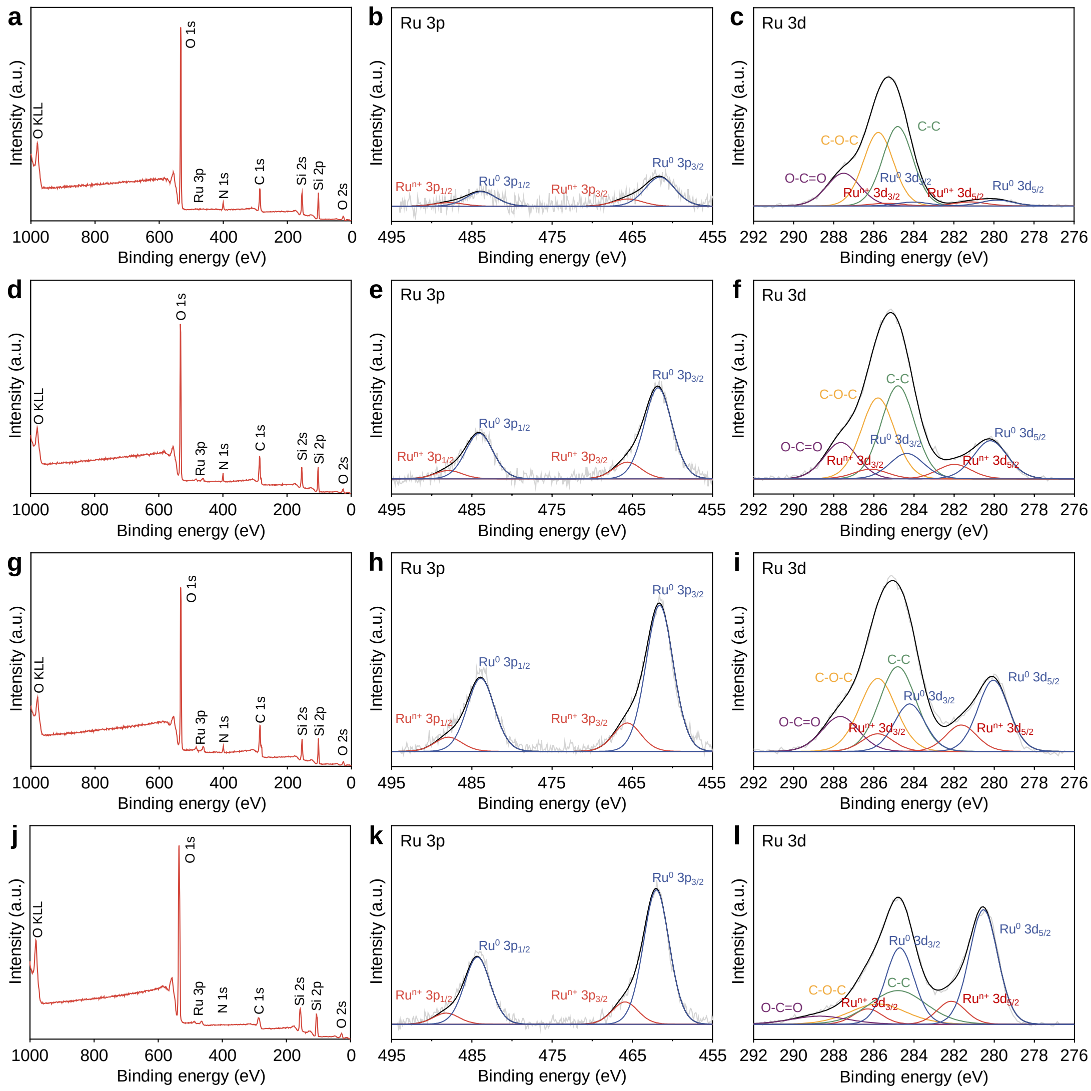

Fig. S20 X-ray photoelectron spectroscopy survey spectra and high-resolution Ru 3p and Ru 3d XPS spectra of Ru/SiO_2 with a different Ru loading or Ru particle size distribution. (a–c) 0.76%-Ru/SiO_2, (d–f) 2.82%-Ru/SiO_2, (g–i) 6.61%-Ru/SiO_2, (j–l) 3.7 nm-Ru/SiO_2.

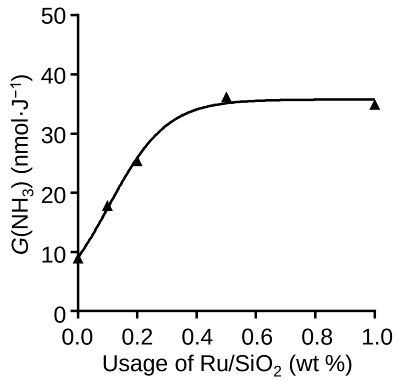

Fig. S21 $G(NH_3)$ in 1 M HCO_2Na solution with 8.0 atm of nitrogen under 3250 Gy of γ-ray irradiation with different usages of Ru/SiO_2.

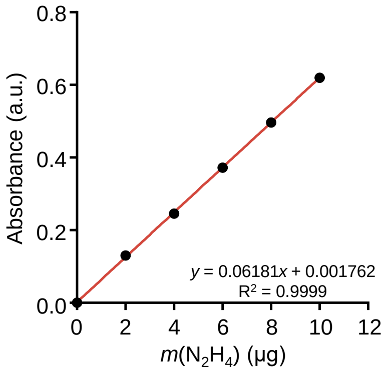

Fig. S22 The calibration curve for quantifying hydrazine via the Watt and Chrisp method.

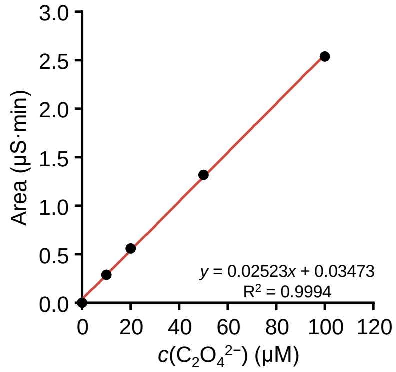

Fig. S23 The calibration curve for quantifying $C_2O_4^{2-}$ via ion chromatography.

Table S1 Determination of $G(\cdot OH)$ in 1 M HCO_2Na solution.

Condition	Fluorescence intensity	$G_{app}(\cdot OH)$ (nmol J^{-1})	$G(\cdot OH)$ (nmol J^{-1})
Ultrapure water	154.4	1.4	280.0
1 M HCO_2Na solution	55.6	0.3	61.0

Table S2 Ru loadings of the commercially available 5% Ru/Al_2O_3 and radiation-synthesized Ru/SiO_2 before and after the reaction.

Sample name	Before the reaction (wt %)	After the reaction (wt %)
5% Ru/Al_2O_3	5.00	3.99
Ru/SiO_2	0.36	0.35
6.61%-Ru/SiO_2	6.61	6.44

Table S3 Ru loadings of radiation-synthesized Ru-based catalysts.

Sample name	Ru loading (wt %)
Ru/γ-Al_2O_3	3.30
Ru/TiO_2	2.84
Ru/CeO_2	2.46
Ru/ZnO	3.81
Ru/SiO_2	0.36
0.76%-Ru/SiO_2	0.76
2.82%-Ru/SiO_2	2.82
6.61%-Ru/SiO_2	6.61
3.7 nm-Ru/SiO_2	7.67

Supplementary references

1. Que X, Li S, Hu Y *et al*. Fluorescence spectroscopy study on the reaction of hydroxyl radicals and hydrated electrons in MXene suspension. *J Phys Chem C* 2022; **126**: 10703–12.
2. Watt GW and Chrisp JD. Spectrophotometric method for determination of hydrazine. *Anal Chem* 1952; **24**: 2006–8.
3. Ross CK, Klassen NV, Shortt KR *et al*. A direct comparison of water calorimetry and Fricke dosimetry. *Phys Med Biol* 1989; **34**: 23–42.
4. Hubbell JH. Photon mass attenuation and energy-absorption coefficients from 1 keV to 20 keV. *Int J Appl Radiat Isot* 1982; **33**: 1269–90.
